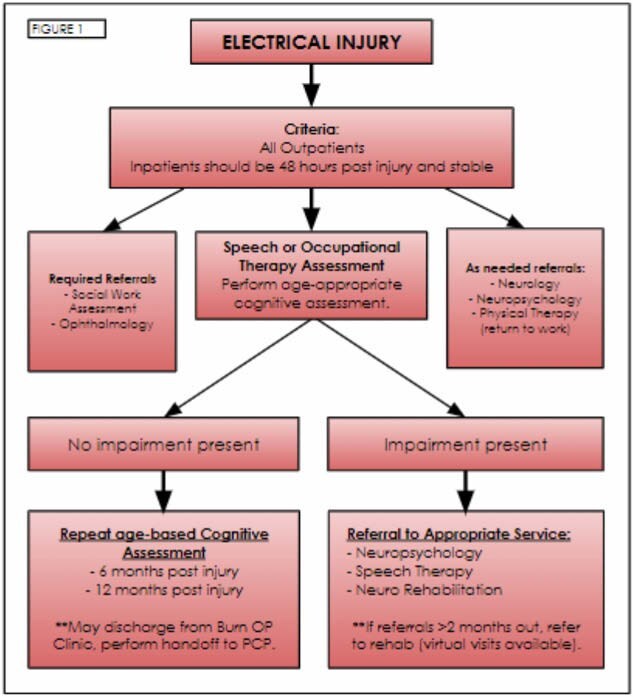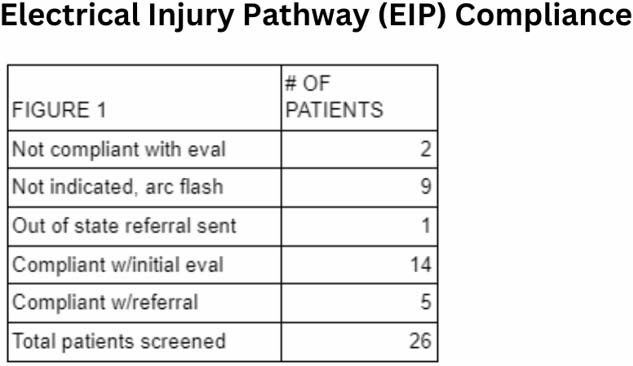# 998 Compliance and General Incidence of Abnormal Cognitive Evaluations from the Electrical Injury Pathway

**DOI:** 10.1093/jbcr/iraf019.529

**Published:** 2025-04-01

**Authors:** Hadley Regal, Callie Thompson, Crystal Webb, Mindy Orr

**Affiliations:** University of Utah Health Burn Center; University of Utah Health Burn Center; University of Utah; University of Utah

## Abstract

**Introduction:**

We created an Electrical Injury Pathway (EIP) in the Fall of 2023 to improve evaluation of cognitive deficits associated with electrical conduction injuries. The EIP expedites access to and treatment of these patients by the speech language pathology (SLP) team. SLP interventions can occur in inpatient or outpatient settings. To assess the efficacy of the EIP, we examined all patients who sustained a conduction injury from 08/01/23 through 08/31/24 who were included in the EIP.

**Methods:**

A retrospective chart review of our burn center patient log was completed from 08/01/23-08/31/24. We identified patients that sustained an electrical injury and met criteria for cognitive evaluation as recommended through the EIP and evaluated their patient, injury, and outcome characteristics.

**Results:**

Chart review indicated 26 patients met criteria for SLP evaluation based on admission order set and mechanism. A comprehensive cognitive evaluation was indicated for 14 patients. Of those 14 patients, 5 patients were found to have abnormal results with the evaluations. Specifically, 2 patients with mild memory/attention deficits, 2 patients with moderate memory/attention deficits, and 1 patient with severe memory/attention deficits. The remaining 9 patients evaluated were found to have cognitive profiles within normal limits. Out of state referral was made for 1 patient and 2 patients were found to not be compliant with evaluation/intervention. Evaluation was not indicated for 9 patients as their injury did not include conduction.

**Conclusions:**

Of the 26 patients screened with the EIP, 12/14 patients received evaluation, indicating 85% compliance with our EIP. Our EIP ensured timely access to, and initiation of, SLP evaluation. Additionally, multidisciplinary communication for our referral process to outgoing facilities was beneficial to our out of state patient who required evaluation. We were able to identify 5 patients who required and received cognitive retraining interventions in both the inpatient and outpatient settings. The EIP resulted in improved performance on follow-up evaluations and allowed for appropriate referrals as indicated for further OP SLP or specialty services.

**Applicability of Research to Practice:**

This workflow was immediately utilized following approval and allowed patients and our care team to understand the typical post injury progression of cognitive function. It provides a pathway to access additional services in the outpatient setting as indicated.

**Funding for the Study:**

N/A